# Risk factors for incidental durotomy during posterior open spine surgery for degenerative diseases in adults: A multicenter observational study

**DOI:** 10.1371/journal.pone.0188038

**Published:** 2017-11-30

**Authors:** Hisatoshi Ishikura, Satoshi Ogihara, Hiroyuki Oka, Toru Maruyama, Hirohiko Inanami, Kota Miyoshi, Ko Matsudaira, Hirotaka Chikuda, Seiichi Azuma, Naohiro Kawamura, Kiyofumi Yamakawa, Nobuhiro Hara, Yasushi Oshima, Jiro Morii, Kazuo Saita, Sakae Tanaka, Takashi Yamazaki

**Affiliations:** 1 Department of Orthopaedic Surgery, Sagamihara National Hospital, Kanagawa, Japan; 2 Department of Orthopaedic Surgery, Saitama Medical Center, Saitama Medical University, Saitama, Japan; 3 Department of Medical Research and Management for Musculoskeletal Pain, 22nd Century Medical and Research Center, Faculty of Medicine, University of Tokyo, Tokyo, Japan; 4 Department of Orthopaedic Surgery, Saitama Rehabilitation Center, Saitama, Japan; 5 Department of Orthopaedic Surgery, Iwai Orthopaedic Medical Hospital, Tokyo, Japan; 6 Department of Orthopaedic Surgery, Yokohama Rosai Hospital, Kanagawa, Japan; 7 Department of Orthopaedic Surgery, Faculty of Medicine, University of Tokyo, Tokyo, Japan; 8 Department of Orthopaedic Surgery, Saitama Red Cross Hospital, Saitama, Japan; 9 Department of Spine and Orthopaedic Surgery, Japanese Red Cross Medical Center, Tokyo, Japan; 10 Department of Orthopaedic Surgery, Tokyo Metropolitan Komagome Hospital, Tokyo, Japan; 11 Department of Orthopaedic Surgery, Musashino Red Cross Hospital, Tokyo, Japan; 12 Department of Orthopaedic Surgery, Sanraku Hospital, Tokyo, Japan; Universita degli Studi di Palermo, ITALY

## Abstract

Incidental durotomy (ID) is a common intraoperative complication of spine surgery. It can lead to persistent cerebrospinal fluid leakage, which may cause serious complications, including severe headache, pseudomeningocele formation, nerve root entrapment, and intracranial hemorrhage. As a result, it contributes to higher healthcare costs and poor patient outcomes. The purpose of this study was to clarify the independent risk factors that can cause ID during posterior open spine surgery for degenerative diseases in adults. We conducted a prospective multicenter study of adult patients who underwent posterior open spine surgery for degenerative diseases at 10 participating hospitals from July 2010 to June 2013. A total of 4,652 consecutive patients were enrolled. We evaluated potential risk factors, including age, sex, body mass index, American Society of Anesthesiologists physical status classification, the presence of diabetes mellitus, the use of hemodialysis, smoking status, steroid intake, location of the surgery, type of operative procedure, and past surgical history in the operated area. A multivariate logistic regression analysis was performed to identify the risk factors associated with ID. The incidence of ID was 8.2% (380/4,652). Corrective vertebral osteotomy and revision surgery were identified as independent risk factors for ID, while cervical surgery and discectomy were identified as factors that independently protected against ID during posterior open spine surgery for degenerative diseases in adults. Therefore, we identified 2 independent risk factors for and 2 protective factors against ID. These results may contribute to making surgeons aware of the risk factors for ID and can be used to counsel patients on the risks and complications associated with open spine surgery.

## Introduction

Incidental durotomy (ID) is one of the most frequent intraoperative complications of spine surgery. The reported incidence of ID ranges from 1.6% to 16% [1–9]. Although many reports have demonstrated good results after surgical repair of durotomies, serious problems secondary to durotomy have also been reported. They include severe headache, pseudomeningocele formation, nerve root entrapment, arachnoiditis, and intracranial hemorrhage [7, 10–12]. As a result, ID can contribute to higher healthcare costs and poor patient outcomes [13, 14].

Previous studies have described the risk factors for ID. They include older age [1, 3, 5–7, 9, 15], female sex [5, 6], experience level of the surgeon [9], elevated surgical invasiveness [3], lumbar surgery [3], revision surgery [1, 3, 15], pre-existing conditions such as degenerative spondylolisthesis [6, 8], ossification of the posterior longitudinal ligament (OPLL) [16], and synovial cysts [6]. However, some of these studies were performed retrospectively, at a single institution, and/or were limited by a small sample size. Even studies with a large sample size were inadequate for examining individual surgical procedures because they used a nationwide database [5, 16]. High-quality studies based on a prospective design and a large sample size are still needed.

The purpose of this study was to clarify the independent risk factors for ID during posterior open spine surgery for degenerative diseases in adults. The study used a prospectively collected multicenter data registry of more than 4,500 patients.

## Materials and methods

### Data source

From July 2010 to June 2013, a multicenter observational study of ID following posterior lumbar spinal surgery in adult patients was conducted in a prospective manner at 10 participating Japanese hospitals. Detailed preoperative and operative information regarding patient demographics, medical comorbidities, surgical procedures, and adverse events were recorded postoperatively through a standardized data collection form. This study was approved by the institutional review boards of Saitama Medical University, Musashino Red Cross Hospital, the University of Tokyo, Yokohama Rosai Hospital, Saitama Red Cross Hospital, Japanese Red Cross Medical Center, Tokyo Metropolitan Komagome Hospital, Sanraku Hospital, Iwai Orthopaedic Medical Hospital, and Sagamihara National Hospital. Because of the observational manner of the study, the institutional review boards of the 10 participating hospitals waived the need for consent from individuals. The opt-out information was available at the following URL (http://www.saitama-med.ac.jp/kawagoe/05others/hec/index.html). The collected patient records and information were anonymized and de-identified prior to analyses.

### Patient population

Patients who underwent posterior open spine surgery for degenerative diseases were included. We excluded patients younger than 20 years of age and those who underwent endoscopic or percutaneous surgery or open surgery for other conditions, such as infection, tumor, and trauma.

### Study measures

The recorded patient characteristics included age, sex, body mass index (BMI), American Society of Anesthesiologists (ASA) physical status classification, presence of diabetes mellitus, the use of hemodialysis, smoking status, steroid intake, location of the surgery (cervical, thoracic, and/or lumbosacral), type of operative procedure (laminectomy/laminoplasty, discectomy, posterior lumbar interbody fusion [PLIF], posterolateral fusion [PLF], and corrective vertebral osteotomy [CVO]), use of instrumentation, and past surgical history in the operated area. We defined “incidental durotomy” as an inadvertent tearing of the dura during surgery with cerebrospinal fluid (CSF) extravasation or bulging of the arachnoid layer.

### Statistical analysis

We analyzed the relationship between ID and potential risk factors. The Student t-test was used to compare the means of the continuous variables between the ID and non-ID groups. For categorical values, the Pearson’s chi-squared test was used to assess the differences in the proportions between the two groups. Relative risks (RRs) and 95% confidence intervals (CIs) were calculated using univariable and multivariable logistic regression analyses. All study variables that have previously been identified as significant risk factors were considered as potential confounders. We entered these variables into the multivariable logistic regression model in order to adjust for potential confounding. The variance inflation factor (VIF) was used to check for multicollinearity in the model. Statistical analysis was performed using SPSS Statistics version 20 (IBM Corporation, Armonk, NY). A *P* value of 0.05 was considered to indicate statistical significance.

## Results

The demographic characteristics of the 4,652 patients included in the study are shown in Table 1.

**Table 1:** Demographic characteristics of the ID group and Non-ID group.

Characteristic	ID group (n = 380)	Non- ID group (n = 4272)	*P* value
Age (years), mean±SD	67.7±12.5	66.0±13.5	<0.01
Male sex, n (%)	196 (51.6)	2608 (61.0)	<0.01
Body mass index (kg/m^2^)	23.9±3.7	24.0±3.7	0.73
ASA score, n (%)			
1 or 2	349 (91.8)	3846 (90.0)	0.24
≥3	31 (8.2)	426 (10.0)	0.24
Diabetes mellitus, n (%)	46 (12.1)	586 (13.7)	0.35
Hemodialysis, n (%)	10 (2.6)	178 (4.2)	0.14
Smoking, n (%)	35 (9.2)	544 (12.7)	0.028
Steroid use, n (%)	6 (1.6)	109 (2.6)	0.23
Anatomic location of the surgery, n (%)			
Cervical	33 (8.7)	947 (22.2)	<0.01
Thoracic	4(1.1)	88 (2.1)	0.18
Lumbosacral	316 (83.2)	3076 (72.0)	<0.01
Operative procedure, n (%)			
Laminectomy/laminoplasty	149 (39.7)	2242 (52.5)	<0.01
Discectomy	23 (6.1)	460 (10.8)	<0.01
PLIF	165 (43.4)	1154 (27.0)	<0.01
PLF	32 (8.4)	385 (8.9)	0.70
CVO	11 (2.9)	33 (0.77)	<0.01
Surgical variables			
Instrumentation	202 (53.2)	1560 (36.5)	<0.01
Revision surgery	87 (22.9)	491 (11.5)	<0.01

ID, incidental durotomy; SD, standard deviation; ASA, American Society of Anesthesiologists; PLIF, posterior lumbar interbody fusion; PLF, posterolateral fusion; CVO, corrective vertebral osteotomy

The total incidence of ID after surgery was 8.2% (380 cases). With respect to demographic characteristics, age, female sex, lumbosacral surgery, PLIF, CVO, and revision surgery have been described as potential risk factors for ID, while smoking, cervical surgery, laminectomy or laminoplasty, and discectomy have been described as potential protective factors. These results are similar to those that we obtained with the univariable logistic regression analysis (Table 2).

**Table 2:** Univariable logistic regression analyses for ID during posterior open spine surgery.

Characteristic	OR (95% CI)	*P* value
Age	1.01 (1.00–1.02)	0.026
Female sex	1.47 (1.19–1.81)	<0.001
Body mass index	0.99 (0.97–1.02)	0.46
ASA score ≥3	0.80 (0.55–1.17)	0.26
Diabetes mellitus	0.87 (0.63–1.19)	0.38
Hemodialysis	0.62 (0.33–1.19)	0.15
Smoking	0.69 (0.49–0.99)	0.046
Steroid intake	0.61 (0.27–1.40)	0.25
Cervical surgery	0.33 (0.23–0.48)	<0.001
Thoracic surgery	0.51 (0.19–1.39)	0.19
Lumbosacral surgery	1.92 (1.46–2.54)	<0.001
Laminectomy/laminoplasty	0.58 0.47–0.72)	<0.001
Discectomy	0.53 (0.35–0.82)	0.004
PLIF	2.08 (1.68–2.57)	<0.001
PLF	0.93 (0.64–1.35)	0.7
CVO	3.83 (1.92–7.64)	<0.001
Instrumentation	1.97 (1.60–2.44)	<0.001
Revision surgery	2.28 (1.77–2.96)	<0.001

ID, incidental durotomy; OR, odds ratio; CI, confidence interval; ASA, American Society of Anesthesiologists; PLIF, posterior lumbar interbody fusion; PLF, posterolateral fusion; CVO, corrective vertebral osteotomy

Table 3 shows the results of the multivariate logistic regression analysis. When we included all of the factors in the multivariate analyses, the VIF value of laminectomy/laminoplasty was 56.6, and the VIF values of discectomy, PLIF, and PLF exceeded 10. This calculation showed multicollinearity between these factors [17]. This multicollinearity is understandable because, in this study, the meaning of “no PLIF nor PLF” and “Laminectomy/laminoplasty” were quite similar.

**Table 3:** Multivariable logistic regression analyses for ID during posterior open spine surgery.

Characteristic	OR (95% CI)	*P* value	VIF value
Age	1.00 (0.99–1.01)	0.82	1.34
Female sex	1.25 (0.998–1.57)	0.052	1.13
Body mass index	0.99 (0.97–1.01)	0.58	1.04
ASA score ≥3	0.93 (0.59–1.43)	0.76	1.38
Diabetes mellitus	0.93 (0.66–1.28)	0.65	1.03
Hemodialysis	0.63 (0.28–1.28)	0.21	1.35
Smoking	0.85 (0.58–1.23)	0.41	1.08
Steroid intake	0.52 (0.20–1.11)	0.10	1.02
Cervical surgery	0.33 (0.18–0.60)	0.0004	5.81
Thoracic surgery	0.38 (0.11–1.05)	0.06	1.52
Lumbosacral surgery	0.78 (0.49–1.28)	0.32	5.92
Discectomy	0.55 (0.33–0.89)	0.01	1.48
PLIF	1.70 (0.91–3.06)	0.09	7.59
PLF	1.05 (0.52–2.02)	0.89	3.59
CVO	3.17 (1.19–7.99)	0.02	1.40
Instrumentation	0.81 (0.46–1.50)	0.50	8.44
Revision surgery	2.04 (1.55–2.67)	<0.0001	1.07

ID, incidental durotomy; OR, odds ratio; CI, confidence interval; ASA, American Society of Anesthesiologists; PLIF, posterior lumbar interbody fusion; PLF, posterolateral fusion; CVO, corrective vertebral osteotomy

Therefore, we excluded laminectomy/laminoplasty from the multivariate analyses. In this model, none of the VIF values exceeded 10, indicating that there was no collinearity in the model [17] (Table 3).

The results suggested that CVO (*P* = 0.02, odds ratio [OR] = 3.17, 95% confidence interval [CI]: 1.19–7.99) and revision surgery (*P*<0.0001, OR = 2.04, 95%CI: 1.55–2.67) were independent risk factors for ID, while cervical surgery (*P* = 0.0004, OR = 0.33, 95%CI: 0.18–0.60) and discectomy (*P* = 0.01, OR = 0.55, 95% CI: 0.33–0.89) were independent protective factors against ID.

## Discussion

In this study, we identified independent risk factors for and protective factors against ID occurring during posterior open spine surgery for degenerative diseases in adults, using a prospective multicenter research study.

Several studies have demonstrated that ID is less likely to occur during cervical surgeries than in thoracic or lumbosacral ones [4, 8]. This result is one that many surgeons can understand. In lumbosacral surgeries, surgeons are more likely to manipulate and retract the dura around the cauda equina instead of the spinal cord itself, which may lead to an increased risk of ID. In terms of anterior cervical surgery, Hannallah reported that ossification of the posterior longitudinal ligament (OPLL) caused the highest risk of ID (13.8 times) because of its adhesion to the dura [18], which are most commonly encountered in anterior approaches to the cervical spine. In this study, we only examined the posterior approach, which diminished the incidence of ID in cervical surgeries.

Discectomy, which is a relatively common and less invasive procedure, was proven to be a protective factor against ID in this study. This result is consistent with those in previous studies that showed that ID was less likely to occur in discectomy than in lumbar spinal decompression [19, 20].

Regarding CVO, this is the first study to evaluate the association between CVO and ID using a prospective multicenter design. CVO is relatively rare surgery; therefore, it is difficult to detect this procedure as a risk factor for ID. This multicenter, large-sample study could have allowed detection of this surgery as a risk factor. CVO involves pedicle subtraction osteotomy and posterior vertebral column resection. In addition to the resection of posterior elements, these procedures also require resection of pedicles, vertebral bodies, and discs, as well as reconstruction of the spinal column with cages and pedicle screw fixation. The high surgical invasiveness and complexity of the procedure may be related to its increased risk of ID.

Many reports have described revision surgery as a significant risk factor for ID [1, 3, 15]. This result is also not altogether surprising for many surgeons. Prior surgeries can cause dural adhesions. Moreover, the absence of normal tissues attributed to prior surgeries can lead to the loss of landmarks during surgery. These atypical conditions could obfuscate anatomy and result in an increased risk of ID.

According to our multivariate analysis, women were 1.25-fold more likely than men to experience ID, and had the tendency to be an independent risk factor (*P* = 0.052, 95%CI: 0.998–1.57). Two previous reports have described female sex as a risk factor for ID [5, 6]; however, neither explained the reasons for this finding. Hong et al. analyzed dural sac thickness in the human spine and concluded that the dural sac tended to be thinner in women than in men [21]. In order to evaluate the association of female sex with ID more precisely, additional high-quality studies with a large sample size are needed.

Knowing these factors associated with increased risk of ID is very important, because they sometimes can cause large lacerations that cannot form sufficiently strong watertight seals, which can lead to severe complications. Khong et al. described a cerebellar hemorrhage caused by ID during PLIF [11]. Ryan et al. described intracranial hemorrhages following ID during pedicle subtraction osteotomy and revision arthrodesis, respectively [10]. Indeed, while repairing the dura after ID is important, being conscious of these risk factors and trying to avoid ID is even more important.

There are several limitations of this study. First, ID in this study only included durotomies detected during the surgery, and therefore did not include cases in which durotomy was speculated based on CSF leakage or severe headache after surgery. Thus, undetected durotomies might have occurred. Second, this study did not account for several factors, such as experience level of the surgeon, spondylolisthesis, or synovial cysts, which were described as risk factors for ID in some studies [9]. In addition, the occurrence of a selection bias during patient enrollment cannot be eradicated. However, we sought to minimize this bias by enrolling consecutive patients from multiple centers, not from a single center.

## Conclusions

In conclusion, this prospective, multicenter study of 4,652 patients used a multivariate analysis and identified CVO and revision surgery as independent risk factors for ID, while cervical surgery and discectomy were shown to be independent factors protecting against ID during posterior open spine surgery for degenerative diseases in adults. By being aware of these risk factors, surgeons could avoid factors leading to ID during surgery. Moreover, surgeons could explain the risks and complications to patients preoperatively.

## Supporting information

S1 File Supporting information. Dataset of this study.
(XLSX)
